# Cyanobacteria: Model Microorganisms and Beyond

**DOI:** 10.3390/microorganisms10040696

**Published:** 2022-03-24

**Authors:** Malihe Mehdizadeh Allaf, Hassan Peerhossaini

**Affiliations:** 1 Mechanics of Active Fluids and Bacterial Physics Lab, Department of Civil and Environmental Engineering, Western University, London, ON N6A 3K7, Canada; mmehdiz@uwo.ca; 2 Department of Mechanical and Materials Engineering, Western University, London, ON N6A 3K7, Canada; 3 Astroparticules & Cosmologie (APC) Lab, CNRS, Université de Paris, 75205 Paris, France

**Keywords:** cyanobacteria, *Synechocystis*, microorganisms, photosynthesis, biofilm, algal bloom, photobioreactor, motility, run and tumble, phototaxis, active fluids, rheology of active suspensions

## Abstract

In this review, the general background is provided on cyanobacteria, including morphology, cell membrane structure, and their photosynthesis pathway. The presence of cyanobacteria in nature, and their industrial applications are discussed, and their production of secondary metabolites are explained. Biofilm formation, as a common feature of microorganisms, is detailed and the role of cell diffusion in bacterial colonization is described. Then, the discussion is narrowed down to cyanobacterium *Synechocystis*, as a lab model microorganism. In this relation, the morphology of *Synechocystis* is discussed and its different elements are detailed. Type IV pili, the complex multi-protein apparatus for motility and cell-cell adhesion in *Synechocystis* is described and the underlying function of its different elements is detailed. The phototaxis behavior of the cells, in response to homogenous or directional illumination, is reported and its relation to the run and tumble statistics of the cells is emphasized. In *Synechocystis* suspensions, there may exist a reciprocal interaction between the cell and the carrying fluid. The effects of shear flow on the growth, doubling per day, biomass production, pigments, and lipid production of *Synechocystis* are reported. Reciprocally, the effects of *Synechocystis* presence and its motility on the rheological properties of cell suspensions are addressed. This review only takes up the general grounds of cyanobacteria and does not get into the detailed biological aspects per se. Thus, it is substantially more comprehensive in that sense than other reviews that have been published in the last two decades. It is also written not only for the researchers in the field, but for those in physics and engineering, who may find it interesting, useful, and related to their own research.

## 1. Cyanobacteria

Cyanobacteria, also formerly called “blue-green algae”, are photosynthetic prokaryotes with ~3500 million years of existence on the planet earth [1,2]. They live in a diverse range of environments, from freshwater and marine [3] to terrestrial ecosystems [4]. Cyanobacteria can tolerate and live in the most extreme habitats including geothermal habitats [5], frozen systems [6], and hypersaline environments [7].

**Figure 1:**
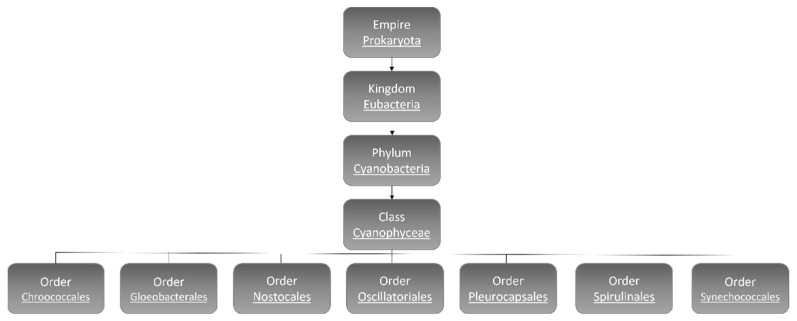
Cyanobacteria classification [9].

The number of cyanobacteria species is still a matter of debate and estimated to reach 8000 [8]. According to morphological characters and molecular analyses, hitherto, 5185 species have been identified and categorized: Chroococcales, Gloeobacterales, Nostocales, Oscillatoriales, Pleurocapsales, Spirulinales, and Synechococcales (Figure 1) [9].

Chroococcales contain coccoid unicelled and colonial cyanobacteria inside a mucilaginous envelope. The famous species in this order is the freshwater bloom-forming species *Microcystis aeruginosa* [10]. Gloeobacterales are unicellular or in irregular groups rod-shaped freshwater cyanobacteria that lack thylakoids [11]. The most identified cyanobacteria are filamentous species that belong to the order of Nostocales, and some species of this order have the ability to fix nitrogen [12,13]. The highest number of benthic linear filamentous species are associated with Oscillatoriales [13] and Pleurocapsales, which can be coccoid cells or resemble filaments (pseudo-filaments), can form complex colony formations [14,15]. Spirulinales members have screw-like coiled filaments while the species categorized in Synechococcales order contain both unicellular (plus colonial) and filamentous types [15,16]. Synechococcales has more than 70 genera and is considered to be the most abundant, ecologically significant, and oldest cyanobacteria. *Synechocystis* sp. is a member of Synechococcales [15,16].

In aquatic habitats, unicellular cyanobacteria are considered as an important group regarding abundance, diversity, and ecological character [15]. Cyanobacteria are also responsible for the primary rise of atmospheric oxygen around 2.3 billion years ago [17] and are known as the key organisms for fixing nitrogen [18]. Recently, cyanobacteria have gained interest in producing bioenergy and valuable biocompounds. Therefore, the development of engineered cyanobacteria has attracted a great deal of attention in the last two decades [15]. One of the most popular single-cell model organisms for genetic, physiological studies of photosynthesis, and energy research is a unicellular freshwater cyanobacterium known as *Synechocystis* [19,20]. The entire genome of *Synechocystis* sp. PCC 6803, as the first phototrophic organism and the fourth organism in general, was completely sequenced in 1996 [19,21].

**Figure 2:**
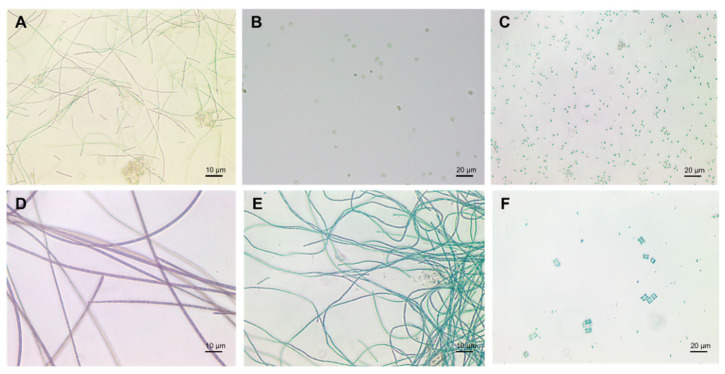
Cyanobacteria: (**A**) *Plecctonema*, (**B**) *Synechocystis*, (**C**) *Microcystis*, (**D**) *Planktothrix*, (**E**) *Anabaena*, (**F**) *Merismopedia*.

Cyanobacteria exist in different morphologies including, unicellular, colonial, and multicellular filamentous forms (Figure 2) [22]. Their cell size varies from less than 1 µm in diameter (Picocyanobacteria) up to 100 µm (some tropical forms in the genus *Oscillatoria*) [3,23,24].

**Figure 3:**
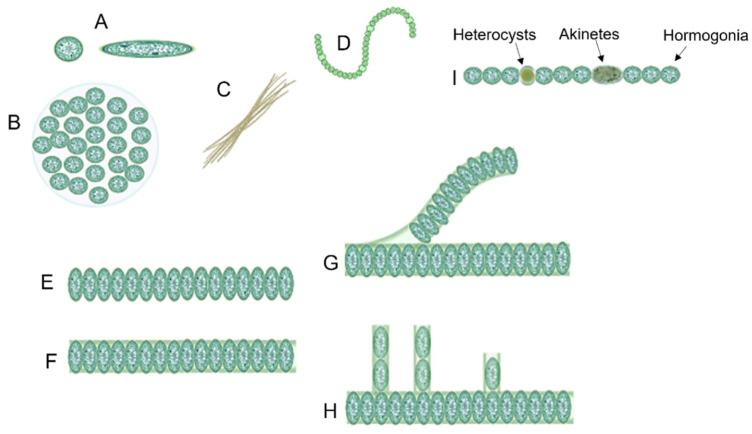
Different forms of cyanobacteria: (**A**) spherical and ovoid unicellular, (**B**) colonial, (**C**) filamentous, (**D**) spiral, (**E**) unsheathed trichome, (**F**) sheathed trichome, (**G**) false branching, (**H**) true branching, (**I**) different cell types in filamentous cyanobacteria. Most parts of this image were created with BioRender (https://biorender.com/).

Unicellular cyanobacteria have spherical, ovoid, or cylindrical cells that may aggregate into irregular or regular colonies bound together by the mucous matrix (mucilage) secreted during the growth of the colony (Figure 3) [13,25]. Based on the species, the number of cells in each colony may vary from two to several thousand [15].

After cell division, in filamentous cyanobacteria, the cells remain adhered to each other and form a chain known as “trichomes”, which can be enveloped in a mucous sheath in some taxa called filaments [13,26]. When trichomes break or fragment within a filament, false branches or “pseudobranches” are formed, which can exhibit in all cyanobacteria orders. However, in some members of Nostocales, cell division occurs perpendicular or obliquely in more than one plane leading to a true branching (Figure 3) [13,15,26].

Three different prevalent developmental cell types may differentiate from filamentous cyanobacteria vegetative cells, including hormogonia (motile cell type), heterocysts (nitrogen-fixing cell type), and akinetes (spore-like cells) (Figure 3) [27,28].

Hormogonia is a short chain of cyanobacterial filaments with gliding motility [29]. In some cyanobacteria species, hormogonia cells contain gas vacuoles to regulate buoyancy in the water column [30]. The major role of hormogonia is in the relocation and symbiotic colonization of hosts [26,30,31,32]. It was reported that the formation of hormogonia can be induced by the exchange of metabolites [33].

Heterocysts are large round-shaped cells with a thicker cell envelope in comparison with vegetative cells that are present in many multicellular cyanobacteria such as *Anabaena* and *Nostoc* and are specialized for fixing nitrogen [30,33].

Akinetes are perennating spore-like cells that can be formed under unfavorable growth conditions such as cold or drought [33]. Like heterocysts, akinetes have a thick cell wall [26]. It was hypothesized that akinetes are the evolutionary precursors of heterocysts [34].

### 1.1. Cyanobacteria’ Characteristics

#### 1.1.1. Cyanobacteria’ Cell Membrane

**Figure 4:**
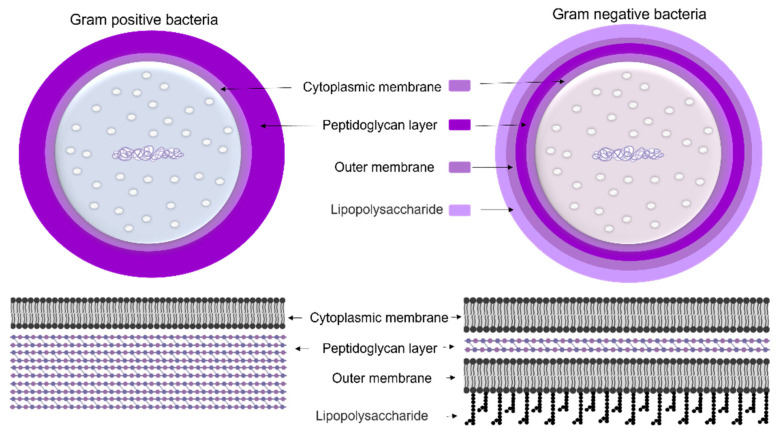
The cell wall composition of Gram-positive and Gram-negative bacteria. Part of this image was created with BioRender (https://biorender.com/).

To protect bacteria from the unpredictable and often hostile environment, their cells are enclosed by a complex multilayered structure. The structure of this layer is not similar for all bacteria and is divided into two major categories known as Gram-positive and Gram-negative, where Gram-positive bacteria are surrounded by a thick layer of peptidoglycan but lack an outer membrane. On the other hand, Gram-negative bacteria have a thin peptidoglycan cell wall, as well as an outer membrane containing lipopolysaccharide (Figure 4) [35].

**Figure 5:**
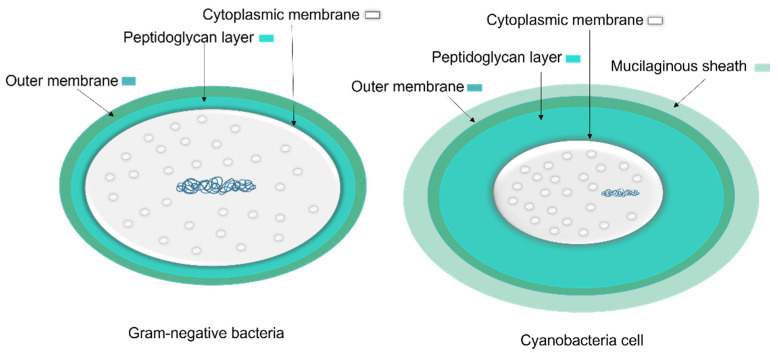
A comparison of general Gram-negative bacteria and cyanobacteria cell envelopes. The peptidoglycan layer in cyanobacteria is thicker than other Gram-negative bacteria and the mucilaginous sheath is present in some taxa.

The cell wall composition of cyanobacteria is a specific group of Gram-negative bacteria [36] with a thin layer of peptidoglycan [37] and lipopolysaccharide outer membrane [38]. However, the cyanobacteria’s peptidoglycan layer is thicker (10–700 nm) compared to most Gram-negative bacteria (2–6 nm) (Figure 5) [37]. The thin peptidoglycan layer was reported in unicellular strains such as *Synechococcus* (~15 nm) [39], while in filamentous strains such as *Oscillatoria princeps* the thickness of this layer was reported more than 700 nm [40]. Aside from protection and sensing environmental stress, transport of nutrients and metabolites into and out of the cell is one of the major roles of the cellular membrane [37].

Similar to Gram-negative bacteria, some cyanobacteria have extracellular non-flagellar appendages, called pili or fimbriae, which involve in motility, adhesion, biofilm formation, and uptake of DNA [41]. The pili are tube-like structures and have been characterized as type IV pili, which are protein polymers. In *Synechocystis* sp. PCC 6803, three different type IV pili were identified after negative staining, including thick pilus with 5–8 nm external diameter and more than 1–2 µm length; thin pilus with 2–4 nm external diameter and 0.5–1 µm length; and a bundle of pili [42,43].

#### 1.1.2. Photosynthesis

Based on the occurrence, abundance, and morphological diversity, cyanobacteria are the largest group of photosynthetic prokaryotes [3], which, like higher plants, are capable of harvesting solar energy and performing photosynthesis through chlorophyll-a by fixing CO_2_ and generating O_2_ [2,44]. Cyanobacteria are responsible for a quarter of global carbon fixation, and suitable engineered photosynthetic microorganisms can increase the capacity of carbon fixation by capturing and storing CO_2_, and thereby stabilize or even reduce atmospheric CO_2_ levels [45].

In addition to chlorophyll-a (green pigment), cyanobacteria produce accessory photosynthetic blue and red pigments known as phycobilin (in particular phycocyanin (PC) and phycoerythrin (PE)), which enable them to grow under low-light conditions. They also produce carotenoids, which are known as a protective agent against photooxidative damages [22,46,47].

Quantitative determination of chlorophylls can be used as a proxy for photosynthesis, primary production, and phototaxonomic studies. However, under starvation, stress, and cell death, these pigments degrade rapidly [48]. Different cyanobacteria species, under various environmental parameters, are capable of producing various levels of these pigments [49].

**Figure 6:**
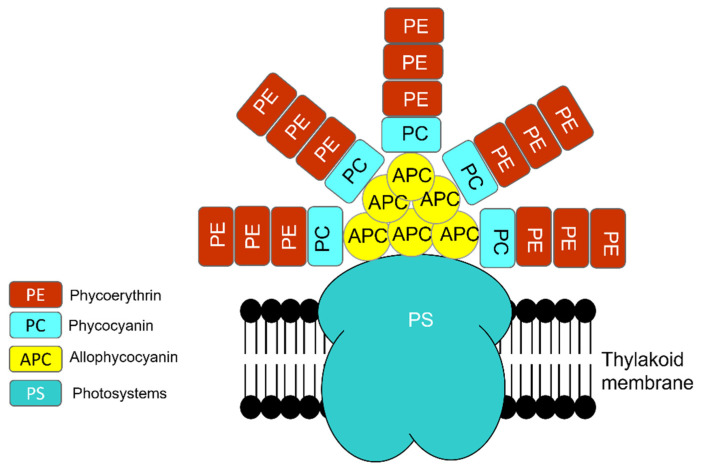
Phycobilisome (PBS) structure is a combination of photosystem (PS), photosynthetic reaction center that contains chlorophyll-a and PC (phycocyanin, A_max_ 620 nm), PE (phycoerythrin, A_max_ 560 nm), and APC (allophycocyanin, A_max_ 650 nm).

Similar to eukaryotic cells, the photosynthetic apparatus of cyanobacteria is made of various units that absorb light energy and produce chemical bond energy such as reduced nicotinamide adenine dinucleotide phosphate (NADPH) and adenosine triphosphate (ATP) [50]. Chlorophyll-a contains reaction centers of photosystem I (PSI) and photosystem II (PSII). However, the major light-harvesting complex (LHC) in cyanobacteria, called the phycobilisome (PBS), is associated with the blue pigmented phycocyanin (A_max_ 620 nm), which absorbs photons in the orange-red part of the light spectrum, and the red-pigmented phycoerythrin (A_max_ 560 nm), which absorbs photons in the green-yellow part of the light spectrum, in conjunction with the PBS core of allophycocyanin (A_max_ 650 nm) (Figure 6) [50,51,52]. Though all PBS contain allophycocyanin and phycocyanin, some contain phycoerythrin [52].

Due to the modification in light intensity and wavelength, temperature, and nutrient availability, multiple regulatory processes evolve to prevent the overexcitation of photosynthesis reaction centers, which lead to the formation of reactive oxygen species (ROS) [53,54]. The produced ROS can damage cellular components and cause cell death; therefore, it is critical to balance light harvesting in the cell [54].

#### 1.1.3. Production of Secondary Metabolites

Aside from the fact that cyanobacteria are mainly oxygen-producing photosynthetic microorganisms, they are also able to develop certain mechanisms to produce a wide variety of secondary metabolites with unique structural features and biological activities in versatile ecological niches including toxins, hormones, iron chelators, antibiotics, antifungal, inflammatory and anti-inflammatory compounds, antimicrobial, antineoplastic, and cytotoxic activities [55,56,57,58,59,60,61,62]. These secondary metabolites, in general, are associated with an organism’s interaction with its environment [63] and probably can be produced to compete with other microorganisms [64]. For instance, the methanolic extract of *Microcystis* showed a significant anticyanobacterial activity against *Anabaena* BT2 and *Nostoc* pbr01 and antialgal activity against a green alga *Bracteacoccus* [58]. Moreover, some of the secondary metabolites are toxic to invertebrates, fish, birds, mammals, and humans [65]. Therefore, to protect the public from exposure to cyanotoxins guidelines values have been introduced by several countries (EU, USA, Canada, Brazil, Australia, South Africa, China, and Japan) [66].

On the other hand, the secondary metabolites can be used in the pharmaceuticals and cosmetics industry as photoprotective, antioxidants, anticancer, etc., which will be discussed more in the application section.

Till now, more than 2000 secondary metabolites have been identified, about 800 of them belonging to marine cyanobacteria. The function of most of these secondary metabolites is usually unknown [67,68].

### 1.2. Cyanobacteria in Nature and Industry

#### 1.2.1. Algal Bloom

The temporal and spatial accumulation of cyanobacteria is known as “blooms” or “algal blooms” [69], which are usually beneficial for aquacultures and wild fisheries operations [70]. However, some algal blooms, known as harmful algal blooms (HABs), can have a negative impact on the aquatic ecosystem, public health, drinking water, recreation, and tourism industry and, therefore, affect the economies [69,70,71]. Climate change, global warming, and eutrophication may increase the frequency and severity of cyanobacteria blooms including the harmful ones in freshwater, estuarine, and marine ecosystems [69,72,73,74]. For instance, 20-year time series (1998–2017) for the Western Basin of Lake Erie showed cyanoHABs have become more severe (larger and longer-lasting) in recent years and accelerated after 2010 [75].

Based on the composition of cyanobacteria communities, specific types of cyanotoxins can be produced [65]. For instance, *Microcystis*, *Planktothrix*, and *Anabaena* are among the species that are able to produce microcystins (MCs) [76] while, *Cylindrospermopsis*, *Aphanizomenon*, *Umezakia*, and *Anabaena* are part of the group of species with the ability to excrete cylindrospermopsin (CYN) [77,78,79]. Nodularins, Anatoxins, Saxitoxins, Lyngbyatoxins, Aplysiatoxins, β-N-methylamino-L-alanine (BMAA), 2,4-diaminobutyric acid (DAB), and Lipopolysaccharides (LPS) are other cyanotoxins produced and excreted by different cyanobacteria species [79]. These toxins are released into the water when cyanobacteria cells die or lysis occurs during harmful algal blooms [80].

#### 1.2.2. Usage of Cyanobacteria

Despite the negative impacts of some cyanobacteria species on the aquatic ecosystem, living organisms and human health, cyanobacteria can be beneficially used in various applications. Cyanobacteria, as the primary photosynthetic organism, can participate in carbon fixation and organic chemical production by converting CO_2_ into biomass and producing carbohydrates, fatty acids, and alcohols as renewable sources of biofuels [81,82].

Biofuels are considered as a renewable energy produced from grown biomass. Based on their production methods, biofuels are divided into three generations. The first-generation of biofuels are derived from edible biomass such as starch, while lignocellulosic materials from nonedible plant biomass are being used as feedstock for the second-generation. Microalgae and cyanobacteria represent the third-generation biofuels [82,83].

Some of the advantages of using cyanobacteria as a feedstock for biofuel production reside in their high growth rates and high biomass production that can be converted to biofuels or their precursors. This type of biomass production does not require arable lands used for crop growing and cultivation [81,84]. Cyanobacteria are capable to produce extensive amounts of lipids especially when the cells are under stress. Cyanobacteria can be easily manipulated genetically to convert the atmospheric carbon into biomass or desired end-products in comparison with eukaryotic microalgae [81,82].

For the mass production of microalgae on an industrial scale, very often open ponds and photobioreactors (PBRs) have been used [79,80]. Open ponds are the oldest and least expensive configurations among the different methods; however, the culture suspension in open ponds is highly susceptible to contamination with other microorganisms, difficult to control temperature fluctuations and evaporative losses, and prone to insufficient mixing and light illumination [85,86,87,88]. The biomass production in PBRs, on the other hand, is more costly from the investment point of view, but this system allows a more controllable temperature and nutrient distribution, pH, light exposure, and contamination control strategies [85,86,87,88,89]. Efficient mixing in the PBR volume can enhance the light exposure of microorganisms in suspension [88,89]. However, it should be mentioned that not all bacterial species can survive under severe shear stress caused by mechanical mixing.

One of the most innovative examples of cultivating cyanobacteria and other types of microorganisms for renewable energies and atmospheric carbon fixation is the utilization of energy-efficient bio-solar façades in green buildings. In this application, the building’s façades are covered with double-walled rectangular PBR panels [90], and the produced biomass in the façade bioreactors can be harvested on top of the building and sent to biorefinery for extraction of value-added chemical molecules [91].

Cyanobacteria can also be used as bioremediation agents to eliminate toxic wastes from contaminated sites including soil, water, wastewater, and sediments [92]. Cyanobacteria are able to degrade or detoxify many gaseous, solid, and liquid recalcitrant pollutants such as assimilate atmospheric nitrogen, remove heavy metals from aquatic ecosystems, and reduce the extra phosphate and nitrate in farmlands [93,94,95,96]. Low cost, eco-friendly nature, high efficacity, and public acceptance are the major advantages of using cyanobacteria for bioremediation [92,96,97,98].

The isolated compounds from various species of cyanobacteria of diverse habitats offer different bioactivities and structural properties with high potentials for drug development [99]. Cyanobacteria by-products express antifungal, antimicrobial, anti-inflammatory, anticoagulant, antimalarial, antiprotozoal, antiviral, antituberculosis, antitumor, immunosuppressant to anticancer, and anti-HIV activities [100,101,102,103,104,105]. For instance, the human lung cancer cell line showed apoptosis after exposure to the extract of *Oscillatoria terebriformis* [106], or the methanol extract of *Anabaena* sp. exhibited antibacterial activity against *Escherichia coli* MTCC-739, *Staphylococcus aureus* MTCC-740, *Bacillus subtilis* MTCC-736, *Bacillus cereus* MTCC-430, *Bacillus pumilus* MTCC-1607 [107], while the isolated compounds from *Tolypothrix byssoidea* EAWAG-195 revealed moderate antifungal activity against *Candida albicans* [108]. It has been reported that the potential extracted bioactive compounds from more than 50% of marine cyanobacteria have the ability to kill cancer cells [109]. C-phycocyanin isolated from *Spirulina platensis* acts as a selective inhibitor of cyclooxygenase-2 (COX-2) with hepatoprotective, anti-inflammatory, and anti-arthritic characteristics [110].

Phycocyanin and phycoerythrin pigments, isolated from cyanobacteria, can be utilized, as natural coloring agents in food, drug, and cosmetic industries to replace synthetic pigments [111]. Chewing gums, jellies, ice creams, and fermented milk products are some foodstuffs in which phycobiliproteins are used as natural food colorants [112]. The water-resistant extract of phycocyanin from *Spirulina* is used in cosmetic products such as eyeliner, eyeshadow, and lipsticks [57]. The market value for phycobiliprotein products was estimated to be more than USD 60 million [113]. Some unusual biomolecules, known as mycosporine-like amino acids (MAAs) and Scytonemin produced by cyanobacteria act as sunscreen agents that protect the skin cells against harmful consequences of UV radiation [114,115,116,117].

In agriculture, cyanobacteria are used as bio-fertilizer to improve soil fertility, agricultural productivity, and serve as available nitrogen sources particularly in rice cultivation in many Asian countries [118,119,120]. Employing cyanobacteria products and biomass is a more sustainable and highly effective method to improve crop production and protection due to their fertilizing, biostimulating, and biopesticide potential [121]. Free fatty acids, polysaccharides, carotenoids, and phytohormones are some cyanobacterial biologically active compounds with potential interest in agricultural practices [121]. Genetically engineered cyanobacteria can be used as multifunctional bioagents for eco-friendly and sustainable agriculture [120].

On the other hand, cyanobacteria contain many essential nutrients including proteins, amino acids, vitamins, minerals, essential fatty acids, and phytonutrients; that are being used as dietary supplements and whole food in many countries around the globe [122]. *Anabaena*, *Nostoc*, and *Spirulina* genera have been consumed as food for centuries [49,123].

## 2. *Synechocystis* sp.: A Model Microorganism

**Figure 7:**
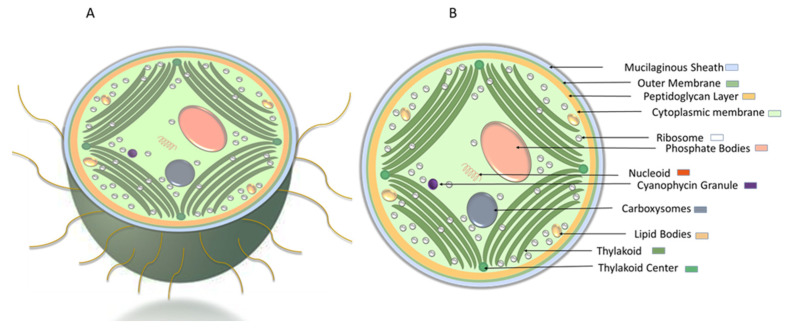
Schematic representation of *Synechocystis* cell morphology. (**A**) cross-sectional view of *Synechocystis* cell with pili. (**B**) ultrastructure of *Synechocystis* cell.

*Synechocystis* sp. is a unicellular, spherical, non-nitrogen-fixing cyanobacterium with 0.7–8 µm in diameter and no or fine and colorless mucilage layer (Figure 7) [19]. The cell envelop of *Synechocystis* sp. contains the outer membrane, a peptidoglycan layer, and cytoplasmic membrane. The thylakoid membranes, derived from the cytoplasmic membrane, cover the peripheral region of the cell [124,125]. From cyanobacteria to higher plants, thylakoid membranes are the site of photosynthesis [126] converged near the cytoplasmic membrane. Thylakoid centers, fibrous coated cylindrical structures, 40–50 nm in diameter and 50–1000 nm in length, establish and maintain thylakoid membrane organization [127,128]. In the cytoplasm, various components such as carboxysomes, ribosomes, polyphosphate bodies, lipid bodies, and cyanophycin granules are detectable [125,128]. In the central cytoplasmic region, carboxysomes improve the carbon fixation by the cell [125,129], while the stored phosphate in polyphosphate bodies can be used for adenosine triphosphate (ATP), phospholipid, and nucleic acid biosynthesis [125].

*Synechocystis* sp. cell’s content is more or less homogeneous and proliferates by binary fission (cleavage). The divided cells can be paired for a short time after division [10,19].

Appendages on the surface of *Synechocystis* sp., called pili, are engaged in motility, adhesion, biofilm formation, and DNA uptake [41]. The pili on the *Synechocystis* sp. surface are identified as type IV (T4P), after genome sequencing [19]. T4Ps are also responsible for suspending the cells and controlling their position in the water column by increasing the cell viscous drag and also the extension/retraction activity [130]. Pili structures are detectable with a scanning electron microscope and transmission electron microscope after negative staining [41].

The entire genome of *Synechocystis* sp. strain PCC 6803, as the first photosynthetic autotroph, was sequenced in 1996 by Kaneko et al. [21]. Due to short generation times, the ease of genetic manipulation, and the limited size of genome and proteome, *Synechocystis* sp. is a suitable model organism to study photosynthesis, lipid metabolism, stress responses, molecular biology, genetic systems, pili system, biofilm formation, etc. [15,19,43,81,131,132]. Genetic manipulation with the aim to improve valuable products has attracted a great deal of attention in the past years; however, it is still in its infancy. In a study on protein–protein interactions (PPIs) it was shown that photosynthesis plays a role in metabolism, cell motility, DNA repair, cell division, and other physiological processes. The additional level of PPIs information greatly enhanced the molecular mechanisms of photosynthesis and therefore improved the understanding of the molecular organization in *Synechocystis* sp. [133]. The mutant strain of *Synechocystis* sp. with a knockout gene (*slr512*) showed a better high light acclimation compared to the wild-type strain [134]. Metabolic engineering design with target genes involved in the process improved the lipid and ethanol productions [135,136] and proliferative growth and certain contents of intracellular pigments including chlorophyll-a and carotenoids [137,138].

### 2.1. Motility

#### 2.1.1. Type IV Pili

Type IV pili are complex multi-protein apparatus with 15–20 kDa pilin subunits, extremely thin (6–9 nm in diameter), and longer than 1 µm, with the ability to form bundles [41,139,140]. After negative staining, three morphologically different type IV pili were identified in *Synechocystis* sp. PCC 6803; thick, thin, and bundle [42,43,141]. Thick morphotype has a length of 1–3 µm and 6–8 nm in diameter, thin morphotype has 0.5–1 µm in length and 2–3 nm in diameter, and a bundle of pili with a diameter less than 45 nm. The thin morphotype covers the entire outer surface of the *Synechocystis* cell, while the thick ones are used to link between cells, and are required for motility and transformation competency [42,141]. Type IV pili play important roles in the twitching motility of cells, biofilm formation, aggregation, adhesion, uptake of DNA, and natural competence or secretion [41].

**Figure 8:**
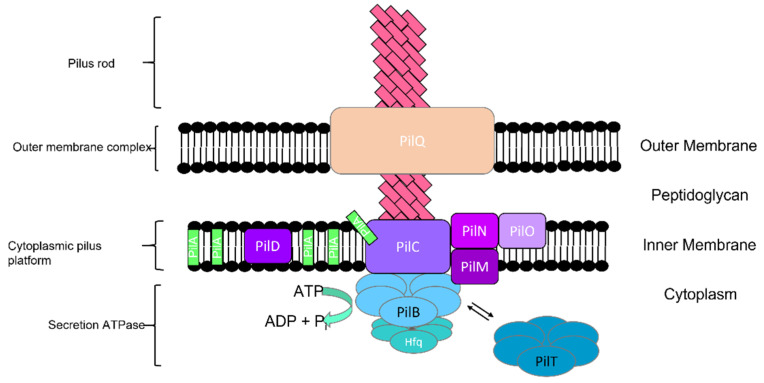
Type IV pilus and four distinct subcomplexes in cyanobacteria. Modified/Adapted with permission from [41].

The pilus rod, outer membrane complex, cytoplasmic pilus platform, and secretion ATPases are four distinct subcomplexes of the type IV pili apparatus (Figure 8) [41]. Pilus is made of 500–1000 major structural component protein units called pilin. In addition to pilin, PilQ, PilN, PilO, PilM, PilC, PilB, PilT, and PilD are other proteins in the type IV pilus system [41,141,142,143]. The transport of pilins across the outer membrane and alignment of the pore complex with the pilus platform is facilitated by the protein structure in the membrane complex and powered by secretion ATPases [41,144]. The motility direction of the *Synechocystis* cell is controlled by the localization of the type IV pili apparatus and strongly correlates with the orientation of the PilB protein adjacent to a specific region of the cytoplasmic membrane (Figure 8) [142,145]. Different studies show two groups of genes: a group of genes that are involved in pilus biogenesis, motility, and assembled proteins [42,43,146,147], and another group that is involved in signal transduction for pilus assembly and phototaxis [147,148,149]. Inactivated genes are involved in pilus biogenesis and abolish cell motility [42].

It was identified that the motility of cyanobacteria is partially controlled by light and several genes that are important for phototaxis [139,150]. The mutant strain of *Synechocystis* sp. with lacking structural and regulatory components that encode pilin resulted in the loss of motility [131,146,151].

#### 2.1.2. Cell Movement

Motility is a way to respond to a changing environment [152]. Twitching or gliding motility has been detected in many Gram-negative bacteria, including cyanobacteria, mediated by type IV pili [42]. Twitching is translocation over a moist surface, which requires an extension, tethering, and then retraction activities of pili [153]. Gliding motility is a smooth motion in a direction parallel to the long axis of the cell that occurs at an interface such as solid–liquid, solid–air, and solid–solid [154]. Gliding has been observed in filamentous cyanobacteria such as *Oscillatoria* and *Phormidium* species [42] while twitching or gliding motility has been detected especially in *Synechocystis* sp. [151].

**Figure 9:**
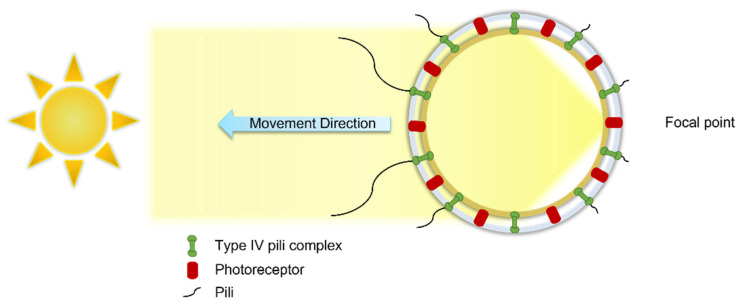
The cyanobacterium *Synechocystis* sp. cell acts as a lens and focuses the light at the opposite side of a light source (focal point). The photoreceptor proteins deactivate the type IV pili complexes near the focal point and activate the ones at the opposite side of the focal point, which leads the cell towards the light source in positive phototaxis. Modified/Adapted with permission from [143,157,160]. Copyright 2022 Elsevier.

The motile behavior of cyanobacteria, including *Synechocystis* sp., can be regulated by the direction, intensity, and wavelength of light, which is known as phototaxis [42,143,155]. Cell size, shape, cell refractive index, and the refractive index of the surrounding medium are the parameters that impact phototaxis [143,156]. In phototaxis, *Synechocystis* cells act as tiny spherical lenses where the image of a light source in the cell’s environment is focused on the cell inner wall. From the focused light, which is detected and analyzed by the light-detecting molecules (called photoreceptors), a signal is sent to the IV pili complexes. Then, the type IV pili complexes, distributed on the cell wall, enable or disable the particular pili for directing the cell towards or away from the light source, leading to a positive or negative phototaxis (Figure 9) [157]. The photosynthetic apparatus also acts as a signaling photoreceptor by absorbing light through chlorophylls, phycobilins, or carotenoids to initiate signal transduction cascades [158]. Red to green wavelengths result in positive phototaxis, while blue wavelength leads to negative phototaxis [159].

The secretion of extracellular substances from the cells improves cell motility, the direction of colony-forming cells, and light focusing by modifying the refractive index near the cell surface [143,155,161]. Single-cell shows limited motility behavior compared to the small colonies [161], by increasing the number of cells in a colony, as a result of cell division or cellular aggregation, the number of motile cells increases [161].

**Figure 10:**
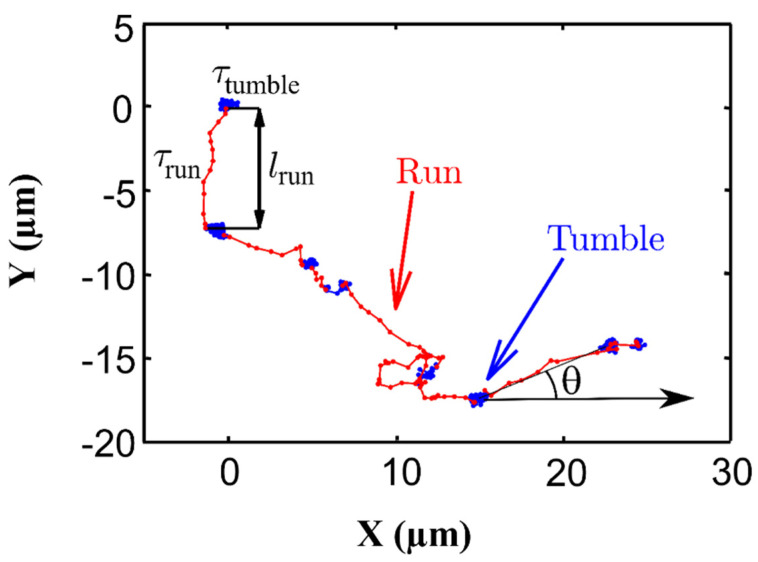
Illustration of run-and-tumble motion of a *Synechocystis* cell extracted from the experimental trajectory of a single cell. During run the cell moves quickly from one point to another, while during tumble it remains constrained in a given area and tends to change directions.

Single *Synechocystis* cells display an intermittent motion in cell suspensions with two phases; a high-motility “run” and a low-motility “tumble” (Figure 10) [150]. The two phases can be modified under various external stressors. Increasing the light intensity, uniformly over the space, increases the probability of *Synechocystis* being in the run state randomly in all directions. This feature, however, vanishes after a typical characteristic time of about 1 h, when the initial probability is recovered. These results were well described by a mathematical model based on the linear response theory proposed by Vourc’h et al. [150].

*Synechocystis* cells can also undergo biased motility under directional illumination. Under directional light flux, *Synehcocystis* cells perform phototactic motility and head toward the light source (in positive phototaxis). Vourc’h et al. [150] showed that this biased motility stems from the averaged displacements during run periods, which is no longer random (as it was in the uniform illumination). They showed the bias is the result of the number of runs, which is greater toward the light source, and not of longer runs in this direction. Brought together, these results suggest distinct pathways for the recognition of light intensity and light direction in this prokaryotic microorganism. This effect can be used in the active control of bacterial flows.

It has also been observed that very strong local illumination inactivates the motility apparatus [157]. Increasing the light intensity of more than ~475 µmol m^−2^ s^−1^ reverses the direction of *Synechocystis* cells to move away from the high levels of radiation source [162,163]. Moreover, *Synechocystis* cells show a negative phototaxis behavior under ultraviolet radiation as an effective escape mechanism to avoid damage to DNA and other cellular components of *Synechocystis* [159,163,164].

Contrary to the run phase that can extend from a fraction of a second to several minutes, the tumble lasts only a fraction of a second [165]. The tumbling phase is a clockwise rotation that allows the cell to change the motility direction of the next run [166,167].

Chemotaxis is another scheme that allows an organism to move toward or away from gradients of nutrients or other chemical stimuli. Detecting by transmembrane chemoreceptors [153,165,168] the microorganism performs a three-dimensional random walk is observed in a homogenous environment, and the direction of each run is identified after a tumble [166].

### 2.2. Biofilm Formation

Bacteria exist predominantly within biofilm in natural, industrial, and clinical settings. Recent research has established that more than 99% of microorganisms in natural settings are fixed on surfaces, due to nutritional and protective benefits linked with life in the adherent populations. In contact with a solid surface, cyanobacteria can aggregate and form a biofilm to grow and survive particularly under environmental stresses [131]. The formation of biofilm by pathogenetic organisms in industrial settings and clinical instruments can infect the living host and can cause chronic infections [169,170]. Alternatively, phototrophic biofilms can be employed in various useful applications such as wastewater purification, bioremediation, aquaculture, and agriculture [96,118,171,172].

Nutrient gradient, cell differentiation, quorum sensing, bacterial motion, and their interaction with the environment can affect the biofilm structure [173]. Microcolonies can form once the bacteria contact the solid surface [174]. Then, once the biofilm developed and matured, some cells secrete chemical molecules that allow the cells to disperse in the environment [175].

**Figure 11:**
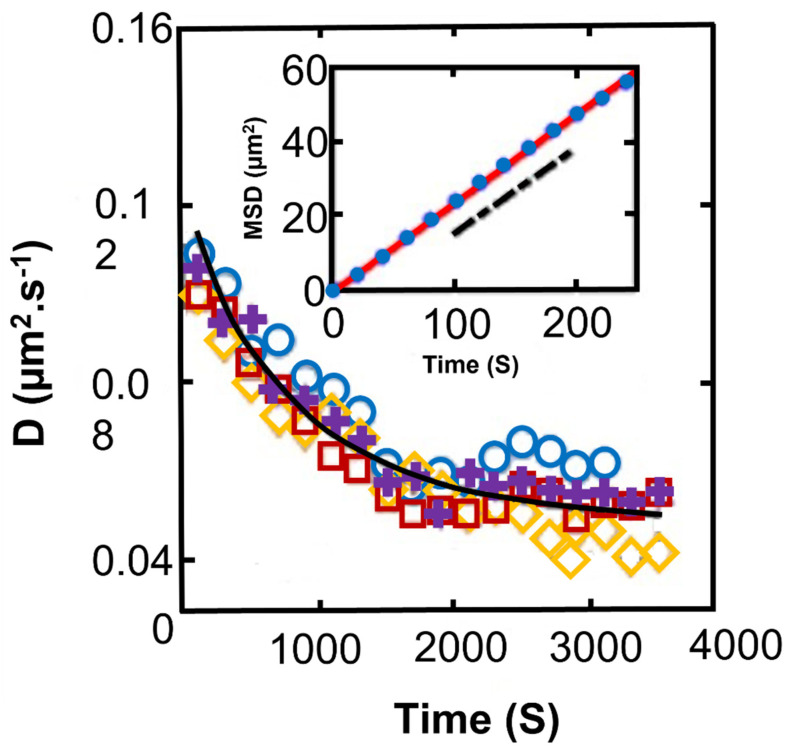
Temporal evolution of the diffusion coefficient of *Synechocystis* for different experiments; black line: a phenomenological model. Inset: experimental MSD at long times (circles) and as computed from numerical simulations (line). The dashed black line indicates the slope given by the phenomenological model [131]. Among different species of bacteria and cyanobacteria, *Synechocystis* sp. is a suitable photosynthetic model organism to study biofilm formation and physical characteristics. At present, *Synechocystis* is probably the only cyanobacterium, which has been investigated in such details, making it an interesting lab model bacterium for biotechnological applications [177]. Modified/Adapted with permission from [131]. Copyright 2022 American Physical Society.

Recent research suggests that in cyanobacteria, signaling by secretion of extracellular polysaccharides reduces the diffusion coefficient with time (Figure 11) [131] and, therefore, enhances the formation of biofilm. The hardness of the substrate surface also can affect the motility and the proportion of the motile cells that in turn impact the shape of microcolonies [176].

On a surface exposed to light, phototrophic microorganisms such as unicellular and filamentous cyanobacteria, green algae, and diatoms, are able to form surface-attached communities, called phototrophic biofilms [90,178]. Biofilms are the most successful form of life on the planet earth and the oldest fossilized phototrophic biofilms date back approximately 3.5 billion years ago [96,179,180].

The morphology of biofilms shows various degrees of porosity in the form of mushroom-like macrocolonies surrounded by water-filled voids. They can be smooth and flat, rough, fluffy, or filamentous. However, all morphologies immobilize biofilm cells and permit very diverse habitats of microorganisms on a small scale [181]. The material properties of the surfaces such as surface charge, hydrophobicity, roughness, topography, and stiffness affect adhesion and biofilm formation [182,183,184].

Biofilm initiation depends on the motility of *Synechocystis* in the first place, followed by physicochemical and electrostatic interactions between the surface and the microorganism’s envelope, and microorganism cells together [173,185,186]. Focusing on the dynamics at the cell scale, the balance between nucleation-division and diffusion-aggregation processes controls the emergence of microcolonies. For example, motility can either favor bacterial aggregation by enabling cell-cell encounters, but also can prevent localized aggregates by enhancing dispersion. The growth of microcolonies of *Synechocystis* on soft and different hard surfaces was studied by Vourc’h et al. [176]. The results showed that soft surfaces promote higher amounts of motile bacteria *p_m_* than the hard ones, and the number of cell clusters at long times was a decreasing function of *p_m_*. Therefore, it was proposed that motility allows the bacteria to escape from clusters while non-motile ones are trapped [176]. This study highlighted that for an adequate description of the biofilm formation of *Synechocystis* it is necessary to account for subpopulations of variable dynamics among a given cell strain. A kinetic model that emphasizes specific interactions between cells, complemented by extensive numerical simulations considering various amounts of cell motility, described adequately the experimental results of this study [176], the high proportion of motile cells enhances dispersion rather than aggregation.

Photosynthetic metabolisms, in general, convert chemical energy to mechanical energy for overcoming the frictional forces between cells and their environment in biofilms [54]. In *Synechocystis*, pili are essential to initiate biofilm formation and adhesion to both biotic and abiotic surfaces. Pili provides intercellular interactions through aggregation and twitching motility in the secondary structure of the biofilm [186].

To hold the biofilm together, microorganisms, such as *Synechocystis* produce extracellular polymeric substances (EPS). EPS forms around 90% of the dry mass of biofilms and is composed of mostly polysaccharides, which are complex polymeric carbohydrates, proteins, nucleic acids, and lipids [173,181]. The extracellular polysaccharides generated by cyanobacteria are unique compared to other bacteria and contain sulfate groups. The sulfated extracellular polysaccharides in *Synechocystis* known as “synechan” and the whole set of genes that regulated synechan biosynthesis and its transcriptional regulation have been recently identified. Synechan is responsible for the buoyancy and floating of cyanobacteria cells. In addition, many sulfated polysaccharides have antiviral, antitumor, or anti-inflammatory characteristics that can be used for human health [187]. EPS provides also the cohesion of biofilms together, immobilizes biofilm cells, mediates the adhesion of the biofilms to the surfaces, creates a three-dimensional polymer network, and provides an external digestive system [181,188]. On water surface or sewage treatment effluents, the produced EPS by *Synechocystis* species protects the cell death induced by TiO_2_ nanoparticles [189]. In the bloom-forming cyanobacteria and the presence of proper nutrients and light intensity, the high photosynthetic activity of cells leads to O_2_ supersaturation, which nucleates into bubbles. These bubbles trapped within the EPS, migrate the biomass upward and aggregate on the surface [190]. In cyanobacteria, the produced EPS improves the soil water-holding capacity, which prevents erosion [191].

In *Synechocystis* species, in addition to EPS, the S-layer (surface layers of bacterial cell walls), as well as pili, assist to attach the biofilms to surfaces [178,192]. However, the presence of an S-layer may be more important for the initial attachment of cell-glass binding than in cell-cell binding, which can be influenced by the pH and ionic strength of the growth medium [178]. *Synechocystis* biofilm formation could be induced or altered by stress responses such as pH, salinity, nutrient level, and osmolarity of the culture. It has been observed that the *Synechocystis* biofilm was initiated for strains subjected to poor nutrient conditions due to the precipitation with calcium in hard water [178]. Previous research showed that the rate of produced EPS in different strains of *Synechocystis* species (PCC 6803 and PCC 6714) is similar and is related to the stage of growth. The EPS extracted from the above two strains constituted of a minimum of 11–12 mono-oses (number of carbons in open-chain monosaccharides with the suffixes “-ose”) with the ability to form various types of polymers, 15–20% (*w*/*w*) uronic derivatives, 10–15% (*w*/*w*) osamies, and 7–8% molar ratio sulfate residues. Around 20–40% of the total weight of EPS is made of proteins [193].

Occasionally, some biofilms have shown vertically laminated multilayered regions ranging from millimeters to several centimeters, known as microbial mats or phototrophic mats [96,194]. These types of niches are dominated by the various groups of cyanobacteria, colorless sulfur bacteria, purple bacteria, and sulfur-reducing bacteria [195,196]. Mats are boosted by the photosynthetic behavior of cyanobacteria as they provide nutrients for other microorganisms as well as physical strength [195].

The undesired development of biofilms in the wrong place and at the wrong time is known as biofouling, which has resulted in a considerable economic loss due to material decay, blockage of flow-through membranes, and cleaning procedures on medical devices, water purification systems, ship hulls, pipelines and reservoirs, and desalination plants [180,197,198]. The biofouling organisms present a higher tolerance against biocides, and disinfectants [180]. However, it has been observed that the antibacterial, antialgal, antifungal, cytotoxic, immunosuppressive, and enzyme inhibiting activities of cyanobacteria metabolites have the ability to prevent biofouling [199].

### 2.3. Synechocystis in Suspensions

In many natural or industrial (photobioreactor) situations, *Synechocystis* cells are suspended in a fluid medium; the suspension is often called “active” or “living” fluid, in which cells act as microstructural elements of the fluid and convert the chemical energy of nutrients into mechanical energy for driving the flow. Therefore, active fluids can develop complex spontaneous motions in the absence of external pressure or velocity gradients. In active fluids flow, there may exist a reciprocal interaction between the cell and the carrying fluid. Fluid mechanics, in conjunction with the microorganism motility, governs the dynamics of active fluids. On the one hand, cell motility can modify the physical and rheological properties of the active fluid; and on the other hand, flow shear can affect cell growth and cell motility. In addition, concentrated populations of cells can modify the influence of the environment that acts on the cell, e.g., by shading from the light or depleting of nutrients. Recent experimental results [200] reveal that shear flow affects the growth rate, doubling per day, biomass yield, pigments and lipid production, and rheological properties of *Synechocystis* sp. CPCC 534 suspensions. The results showed a significant increase in biomass, doubling per day, yield production, and the total amount of chlorophyll-a and carotenoid due to the induced shear flow in comparison with the non-sheared suspension [200]. Meanwhile, shear showed a negative impact on lipid production and cell size [200]. In addition, it is shown the mixing method by which shear has been imposed on the flow can directly impact the growth and pigment production of *Synechocystis* sp. [201,202]. The growth and pigment production were higher in cultures that were grown under turbulent mixing compared to orbitally shaking [201]. In addition, the growth and pigment production presented an improvement in an agitated photobioreactor (turbulent mixing) and airlift bubble column photobioreactor compared to the stationary culture [202]. The highest level of cellular pigments were detected in the early stages of cultural growth when the cells were preparing for the rapid growth phase [202].

The rheological behavior of active fluids, in terms of the viscosity of *Synechocystis* cell suspensions, which experienced various shear rates during growth, was also studied [200]. It was observed that the suspension viscosity, normalized at a constant bio-volume fraction of *Synechocystis*, showed Newtonian behavior (viscosity independent from shear stress) at all cell concentrations. This behavior was observed for both pre-sheared and non-sheared samples, implying that the shear history does not have an influence on the rheological behavior of *Synechocystis* suspensions [200]. Concerning the effect of cell motility on viscosity, the experiments showed no significant difference between the viscosity of live-cell and dead-cell suspensions [200]. This behavior was attributed to the low and twitching nature of the *Synechocystis* motility, in contrast to the high motility of swimmers such as *Chlamydomonas reinhardtii*. However, *Synechocystis* concentration showed a noticeable increase in the viscosity of cell suspensions. It was observed that the viscosity is a linearly increasing function of the cell volume fraction, as is for passive rigid particles; and a correlation was proposed for this variation. However, the viscosity increase in *Synechocystis* suspensions was smaller than the one observed in suspensions of rigid spherical particles of similar size [200]. The smaller observed increase in viscosity with cell volume fraction was attributed to the fact that the elastic soft suspended particles (*Synechocystis* cells) can deform; thus, some of the energy of the flow is dissipated in deforming the cells. This decreases the amount of energy dissipated by hydrodynamic interactions compared to rigid particles, so the viscosity is less than it would be for rigid particles. Another (or concomitant) cause can be the effect of the shape dynamics of soft elastic particles as is discussed in Gao et al. [203].

## 3. Summary

Cyanobacteria, also known as “blue-green algae”, are one of the oldest photosynthetic prokaryotes on planet earth, with the ability to live and flourish in a diverse range of environments from hot springs to underneath of ice pack in frozen lacks, and under the surfaces of rocks in deserts. Based on morphology, cyanobacteria can be categorized as unicellular, colonial, or multicellular filamentous; their cell size can vary from 1 to 100 µm in diameter. To protect the cell from unpredictable and often hostile environments, the cells are covered in a complex multilayered structure of peptidoglycan and lipopolysaccharide outer membrane. In colonial form, mucous matrix (mucilage) may be secreted during the colony growth to bind the cells together.

Cyanobacteria, like higher plants, are capable of converting light energy into chemical energy and generate O_2_. For this purpose, they use different types of pigments including chlorophyll-a (green pigment), phycobilins such as phycocyanin (PC) and phycoerythrin (PE), as well as carotenoids. In addition to oxygen and chemical energy, and as a result of the interaction of microorganisms with their environment, cyanobacteria can produce a wide variety of secondary metabolites with unique biological activities. Toxins are one of them. In the aquatic ecosystem, cyanobacteria overgrowth, with the ability to produce toxins, can be triggered by high temperature and abundant nutrients and, therefore, lead to the formation of harmful algal blooms (HABs). HABs have adverse effects on the aquatic ecosystem, public health, drinking water, recreation, and the tourism industry.

Cyanobacteria also have positive impacts on the environment and economy. Some cyanobacteria can be used in applications such as feedstock for biofuel production, bioremediation agents to eliminate toxic wastes from contaminated sites including soil, water, wastewater, and sediments, bio-fertilizers to improve soil fertility in agriculture, supplement for animal and aquacultural feed, as well as human nutrition, and also in the pharmaceutical, food, and cosmetic industries.

There are still many open questions regarding different physioecological characteristics of cyanobacteria that require further investigation, such as mechanistic details of photosynthetic and respiratory electron transport, important metabolic reactions, biofilm signal transduction pathway, the impact of climate change and global warming on ecological and biogeochemical qualities of cyanobacteria, dominant species and the mechanisms that maintain their dominance, HABs formation, toxins production and activation mechanisms, and more.

Among different cyanobacteria, a unicellular, spherical, non-nitrogen-fixing cyanobacterium, *Synechocystis* sp., occupies a special place in phycology. Its entire genome, as the first photosynthetic microorganism, was sequenced in 1996. *Synechocystis* sp. has been used as a model organism to study many biological and dynamical aspects of cells, from photosynthesis to biofilm formation.

*Synechocystis* sp. is a small, 0.7–8 µm prokaryotic microorganism, whose outer surface is covered with complex multi-protein apparatus known as type IV pili. The type IV pili are engaged in motility, adhesion, biofilm formation, aggregation, DNA uptake, and natural competence or secretion. Twitching and gliding movements are both identified in *Synechocystis* sp. Twitching occurs at a moist solid surface, while gliding is relocation at interfaces such as solid–liquid, solid–air, and solid–solid. *Synechocystis* sp. moves on a random-walk shaped trajectory composed of two phases; a “run” phase with high motility and a “tumble” phase with low motility. Environmental stressors such as light can increase the probability of a “run” state. Direction, intensity, and wavelength of light and the presence of favorable chemicals and nutrient gradients can directly impact the migration and movement of *Synechocystis* sp. cells.

Motility of *Synechocystis* is the first factor for biofilm initiation and formation on both biotic and abiotic surfaces, followed by physicochemical and electrostatic interactions between the surface and the microorganism’s envelope, and microorganism cells together.

The physical and rheological properties of the suspension of *Synechocystis* sp. can be affected by cell motility and concentration. The viscosity of *Synechocystis* with various cell concentrations at a constant bio-volume fraction revealed a Newtonian behavior while the viscosity is a linearly increasing function of the cell volume fraction.
